# Abdominal tuberculosis: sonographic diagnosis and treatment response in HIV-positive adults in rural South Africa^☆^

**DOI:** 10.1016/j.ijid.2009.11.030

**Published:** 2010-09

**Authors:** Tom Heller, Sam Goblirsch, Claudia Wallrauch, Richard Lessells, Enrico Brunetti

**Affiliations:** a Hlabisa Hospital, Hlabisa, KwaZulu-Natal, South Africa; b Department of Medicine – Pediatrics, University of Minnesota, Minnesota, USA; c Africa Centre for Health and Population Studies, University of KwaZulu-Natal, PO Box 198, Somkhele, Mtubatuba 3935, KwaZulu-Natal, South Africa; d Division of Infectious and Tropical Diseases, University of Pavia, IRCCS S. Matteo Hospital Foundation, Pavia, Italy

**Keywords:** Ultrasound, TB, HIV

## Abstract

**Objective:**

To investigate the diagnostic value of abdominal ultrasound in HIV-positive inpatients in a rural African setting.

**Methods:**

This was a prospective case series over 3 months of adult HIV-positive patients with symptoms suggestive of abdominal tuberculosis (TB). Diagnostic ultrasound was performed for all patients: sonographic criteria included abdominal lymph node enlargement (>1.5 cm) and focal splenic lesions; ascites was a supportive finding. Further diagnostic studies, e.g., aspiration or biopsy were not routinely performed. TB treatment was initiated on the basis of clinical and sonographic features. The patients were contacted after 4 months to evaluate the clinical outcome.

**Results:**

One hundred and eighty adult HIV-positive patients were screened; 30 (16.7%) showed sonographic signs of abdominal TB. The median CD4 count was 78 cells/mm^3^. Presenting symptoms were weight loss (86.7%), abdominal pain (76.7%), and diarrhea (60%). Abdominal lymph node enlargement was the diagnostic finding in almost all cases (96.7%); hypoechoic lesions of the spleen were seen in 50% and ascites in 73.3%. Follow-up information was available for 25 patients: 24% had died and the remaining 76% reported symptomatic improvement and weight gain.

**Conclusions:**

Characteristic sonographic features of abdominal TB are common in HIV-infected inpatients in a rural African setting. Ultrasound should be introduced into clinical algorithms for the diagnosis of extrapulmonary TB.

## 1 Introduction

The convergence of the HIV and tuberculosis (TB) epidemics in Southern Africa has contributed to a significant increase in reported cases of TB and especially of extrapulmonary TB (EPTB).^1^ Abdominal TB is a frequent localization of EPTB in HIV-positive patients.^2^ Abdominal symptoms are common at all stages of HIV infection, but particularly at advanced stages of immunosuppression. It can be challenging in a setting with limited diagnostics to differentiate between symptoms caused by HIV itself and symptoms related to opportunistic infection or neoplasm.^3^ The lack of advanced imaging techniques such as computed tomography (CT) and magnetic resonance imaging (MRI), and the limited use of cytology, histology, and cultures, compel physicians to make treatment decisions based on history, clinical examination, and basic laboratory tests. Simple non-invasive diagnostic tools are urgently required in resource-limited rural settings. In the rural district hospital setting, ultrasound is often the most commonly available imaging modality.

World Health Organization guidelines contain criteria for the diagnosis of some forms of EPTB, but do not explicitly include abdominal TB.^4^ Typical ultrasound findings of abdominal TB have been described in low HIV prevalence settings.^5–7^ They include retroperitoneal and mesenteric lymphadenopathy with node diameter greater than 1.5 cm, multiple splenic hypoechoic nodules between 0.5 cm and 1 cm, and patterns of ascites. Hepatic hyperechoic nodules, retroperitoneal abscess, and bowel wall thickening are less frequently described.^5^ Although similar ultrasound findings of lymphadenopathy and splenomegaly are described in African HIV patients, the role of TB co-infection has rarely been explored.^8,9^ One recent report from Zambia described typical findings of abdominal TB in a high proportion of individuals with low CD4 counts and abdominal symptoms.^10^ We sought to investigate the role of ultrasound in the diagnosis of abdominal TB in a rural district hospital in an area of high HIV and TB prevalence.

## 2 Methods

### 2.1 Study setting

Hlabisa Hospital and its 16 primary healthcare (PHC) clinics serve the Hlabisa sub-district in northern KwaZulu-Natal, South Africa. In an area of approximately 1400 km^2^ lives a mainly Zulu-speaking population of approximately 228 000. The sub-district is predominantly rural with one peri-urban area.

The hospital has 300 beds and is staffed by nine to 12 physicians. Approximately 60 000 outpatient visits and 6500 admissions with an average length of stay of six days occur per year. Care for internal medicine patients is delivered in separate male, female, and TB wards. The diagnostic services available are limited. X-ray and ultrasound can be used as imaging modalities. Basic hematology and biochemistry are available in the hospital laboratory; in addition microscopy of urine and cerebrospinal fluid, TB microscopy, and CD4 counts are done. TB culture and histology are available at the provincial reference hospital, but results rarely influence acute clinical management. Patients can be referred to the provincial hospital for CT and MRI scans, but the distance of approximately 100 km, as well as the limited appointments available, restrict this option to selected cases.

The HIV prevalence in the adult resident population (aged 15–49 years) is 21.5%.^11^ The TB notification rate in Hlabisa sub-district has increased from approximately 700 per 100 000 persons in 2003 to 1700 per 100 000 persons in 2008; the number of reported cases of EPTB has risen over 10-fold from 41 to 522 in the same time-period (personal communication, Department of Health, Umkhanyakude District). The HIV co-infection rate for TB patients is approximately 76%.^12^ The TB Control Programme adheres to national guidelines: treatment is initiated by nurses for smear-positive pulmonary disease; the diagnosis of smear-negative pulmonary or EPTB is made by a physician on the basis of clinical or radiological findings (rarely with culture or histological confirmation).^13^ This occurs in hospital, at the central TB clinic, or by physicians serving the decentralized HIV treatment and care program. Patients can be referred to hospital from the clinics for further diagnostic assessment if required.

### 2.2 Study procedure

Patients were enrolled into the study from June to August 2008. All participants were adult (≥18 years) HIV-positive patients admitted to the general medical wards. Patients from the tuberculosis ward were not included as they were invariably known TB cases admitted to the ward to receive streptomycin injections or multidrug-resistant TB treatment. Patients were initially seen by admitting hospital staff and were referred to the study if they reported one or more of the following clinical symptoms: abdominal pain, abdominal distension, diarrhea, or weight loss. Patients were subsequently interviewed and examined by the study physician assisted by a nurse (who also translated from isiZulu). A standardized data sheet was used to collect patient information, including clinical symptoms, laboratory results, chest X-ray (CXR) results, CD4 cell count, and history of antiretroviral therapy (ART) use. Patients were included if their HIV diagnosis was known at the time of hospital admission or if the diagnosis was made during the inpatient episode.

There is no established ultrasound department at the study hospital and all exams were performed by the study physician using a Toshiba Just Vision 400 Model SSA-325A with a 3.5 MHz abdominal probe in a room adjacent to the X-ray facilities. All ultrasound studies were performed by a physician trained in abdominal ultrasound with more than 10 years of experience in the technique. Abdominal sonographic findings were then recorded on the data sheet.

Abdominal TB was diagnosed on the basis of typical findings of lymph node enlargement greater than 1.5 cm and/or focal splenic lesions. Ascites was noted as further supportive evidence. If only ascites was present this was not considered diagnostic and the patient was not included in the study group; further investigations for alternative diagnoses (e.g., cirrhosis, congestive heart failure) were undertaken. After TB diagnosis, patients were treated according to the South African national TB guidelines:^13^ initial treatment for new cases includes rifampin, isoniazid, pyrazinamide and ethambutol for 2 months, followed by rifampin and isoniazid for 4 months.

If patients were not already on ART they were offered treatment according to South African national guidelines (EPTB is a stage 4 condition and ART is indicated regardless of CD4 cell count).^14^ The first-line ART regimen includes stavudine, lamivudine, and either efavirenz or nevirapine (efavirenz preferred in TB co-infection). Follow-up information was obtained at approximately 4 months by the TB coordinator who contacted the patient by phone and asked a series of questions regarding weight gain, resolution of symptoms (abdominal pain, diarrhea), and whether or not ART had been commenced.

Ethical approval was obtained from the Hlabisa Hospital Ethics Committee. Verbal consent was obtained from all patients; this was considered adequate as all procedures were considered part of routine clinical care.

## 3 Results

One hundred and eighty adult HIV-positive patients with symptoms were screened; amongst these, 30 (16.7%) fulfilled the case definition of abdominal TB based on sonographic features. Patients included in the analysis were between 18 and 49 years of age and 53% were women. The median CD4 count was 78 cells/mm^3^ (91% with CD4 <200 cells/mm^3^; 41% with CD4 <50 cells/mm^3^). Three patients were on ART at the time of the examination (for 1 week, 1 month, and 3 months, respectively). The median body-mass index was 19.9 kg/m^2^ (range 13.3–34.7) (Table 1). The clinical symptoms of the patients are summarized in Table 2; 86.7% presented with weight loss, 76.7% with abdominal pain, and 60% with diarrhea. Laboratory data from the cases at the time of hospital admission are displayed in Table 3.

**Figure 1:**
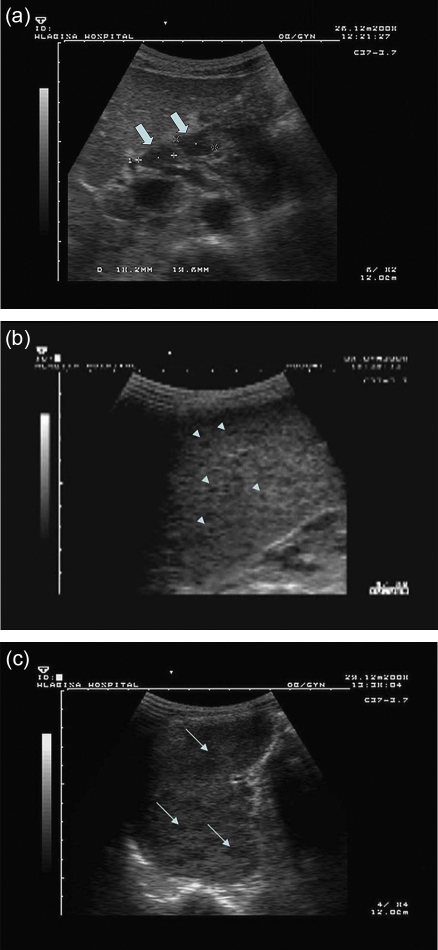
Sonographic image of (a) enlarged lymph nodes in the periportal area; (b) focal lesions in the spleen of approximately 3 mm in diameter; (c) focal lesions in the spleen of approximately 10 mm in diameter.

Abdominal lymph node enlargement was a common finding, present in almost all cases (29/30, 96.7%). Hypoechoic lesions of the spleen were seen in 15/30 (50%) and ascites in 22/30 (73.3%) of the patients. The combinations of findings are shown in Table 4. Examples of abdominal lymphadenopathy and microabscesses in the spleen are shown in Figure 1.

A CXR was available for assessment from 23 patients: 18 (78.3%) demonstrated findings compatible with TB, including five (21.7%) that displayed a miliary pattern; five (21.7%) had a normal CXR.

Telephonic follow-up information was available for 25 patients a mean period of 17 weeks (range 12–23 weeks) after starting TB treatment. Six patients had died (24%), and the median time between start of TB treatment and death was 2 weeks (range 0–17 weeks). The remaining 19 patients (76%) were still taking TB treatment and reported clinical improvement and weight gain. All but one of the 16 surviving patients not already on ART had started this by the time of follow-up.

## 4 Discussion

Ultrasound is safe, portable, inexpensive, non-invasive, and can investigate most organs affected in HIV-infected patients.^15^ These features are critical for investigation of patients in resource-limited settings compared to patients in industrialized countries with a preponderance of CT and MRI scanners, and with trained radiologists to guide the interpretation. Sonographic findings suggestive of abdominal TB, especially in HIV-infected patients, have been described in patient populations of different racial backgrounds and have been suggested as part of the work-up in HIV-infected patients with fever of unknown origin.^5–7,16^ Despite the fact that suggestive findings have been reported in large ultrasound series in African patients, it is unclear whether in the face of other causes of lymphadenopathy the findings are specific enough to be considered diagnostic.^8,9^ The common dilemma for the physician faced with a heavily immunosuppressed patient in this setting is whether there is evidence of TB and a need for treatment or whether to start ART. This is particularly challenging as almost one in four patients still initiate ART with a CD4 count <50 cells/mm^3^.^17^ Commencement of ART without adequate exclusion of TB risks the development of immune reconstitution inflammatory syndrome (IRIS) with consequent morbidity and mortality.^18^

In our patient series we found that ultrasound is a feasible technique to add weight to a diagnosis of abdominal TB in HIV-infected patients. Most patients have severe immunosuppression and report symptoms of weight loss, abdominal pain, and diarrhea, or a combination thereof. The pre-test probability for TB is often relatively high given the extremely high TB incidence in this community. Laboratory investigations in our patients frequently show anemia, most probably anemia of chronic disease related to both HIV and TB. All patients had a reduced albumin, most probably secondary to malnutrition and possibly malabsorption due to bowel disease. Bilirubin and gamma-glutamyl transferase were elevated in a large proportion of patients, suggesting involvement of the liver either as diffuse infiltration or with obstructive disease of the small bile ducts (no visible biliary dilatation was found in the ultrasound exams).

In our series, scanning was made easier by the high proportion of underweight patients, as interference by fat and air is minimal. Abdominal TB was diagnosed by ultrasound without further invasive diagnostic steps; treatment was started on the basis of the imaging findings alone. In the majority of patients the approach led to a favorable outcome suggested by weight gain and cessation of symptoms. Of the six patients who died, three died within a week of the ultrasound exam, suggesting they died due to the severity of their disease. It is interesting to note that three patients were on ART at the time of the diagnosis. All of them started within a short period before the examination, suggesting IRIS as a potential contributory factor unmasking underlying abdominal TB.

Limitations of our study include the lack of microbiological or histological confirmation of abdominal TB. Fine-needle aspiration biopsies are restricted for suspected drug-resistant cases and for clinical cases that show atypical signs (e.g., solid organ infiltration suggestive of lymphoma). Unfortunately, biopsy needles are scarce and histology and TB culture are not performed on site, but rather are sent to the central histology laboratory in Durban, and results rarely contribute to acute management. One has to consider other causes of abdominal lymphadenopathy. HIV itself can cause abdominal lymphadenopathy throughout the continuum of HIV infection, but this is not usually accompanied by other features.^19^ Kaposi's sarcoma (KS) is prevalent in the patient population in Hlabisa sub-district, although most patients with disseminated visceral KS have significant skin involvement, which none of our patient group exhibited. Lymphoma is an additional neoplasm that needs to be considered in the differential diagnosis. Infections with non-tuberculous mycobacteria (NTM) cannot be ruled out as a cause, but they are rarely diagnosed due to a lack of mycobacterial blood cultures; the prevalence of NTM disease in this setting is therefore unknown. Nevertheless, in a series of 31 patients, NTM were reported to cause abdominal adenopathy less frequently than *Mycobacterium tuberculosis*, and splenic microabscesses were not seen with NTM.^20^

We included only patients with symptoms related to the abdomen and did not include patients with isolated fever of unknown origin, so there is a possibility we may have missed additional cases of abdominal TB, particularly those at an earlier, less symptomatic stage. Our exclusion of individuals with ascites alone was based on our experience in this setting that ascites without fibrinous strands and with no other diagnostic signs can usually be attributed to chronic heart failure or liver failure. We may have misclassified some patients by this exclusion, and ascitic fluid culture for *Mycobacterium tuberculosis* is not routinely practiced here due to the cost and the relatively low yield (less than 20% in most series^10^). Our results are also hampered by the lack of information on those screened but not diagnosed with abdominal TB. We lost five out of our 30 patients to follow-up, which is most likely due to communication difficulties and patients with a lack of resources to be able to afford either a cell or land-based telephone.

Further research is needed to assess the impact of a standardized ultrasound screening as part of work-up before starting ART, especially for those with advanced immunosuppression. It would further be interesting to evaluate, if in a fraction of patients the findings of abdominal TB would render CXR unnecessary. Portable ultrasound machines make it possible to examine patients in the PHC clinic setting, which would reduce the need (and the expenses) of patients traveling to hospital for CXR. As trained sonographers are scarce in the resource-limited setting, the possibility of training a wider range of medical doctors or clinical assistants in rural hospitals in a limited and focused assessment with sonography, similar to the FAST technique (focused abdominal sonography for trauma) widely used in emergency medicine, should be investigated.^21^ Preliminary results of a training course around focused assessment with sonography for HIV/TB (‘FASH’) have been reported by our group.^22^ This research into the use of ultrasound obviously does not obviate the need for improved TB diagnostics, particularly point-of-care diagnostics.

## 5 Conclusions

Abdominal ultrasound is an effective diagnostic tool to identify lymphadenopathy and focal splenic lesions suggestive of abdominal TB in HIV-infected patients. As a diagnostic gold standard is not available, the sensitivity and specificity of the method are difficult to determine. Nevertheless, our patient series suggests that ultrasound-based diagnosis followed by TB treatment benefits the vast majority of patients and is a feasible approach when resources for further invasive diagnostics are limited. If implemented on a broader scale, ultrasound would assist physicians and other healthcare workers in making more appropriate treatment decisions instead of having to use the ‘treat first, think later’ approach, thus having the potential to save precious time and resources. Ultrasound has been shown to be a useful and financially affordable adjunct to clinical diagnosis in a wide variety of pathologies at the district healthcare level.^23^ Abdominal ultrasound should be introduced into clinical algorithms for the diagnosis of EPTB.

## Figures and Tables

**Table 1:** Characteristics of patients with abdominal TB

Sex	
Female	53%
Male	47%
Age, mean ± SD, years	31 ± 8

CD4	
Median (range), cells/mm^3^	78 (6–408)
<200 cells/mm^3^	91% (95% CI 81–100%)
<50 cells/mm^3^	41% (95% CI 23–59%)
BMI, median (range), kg/m^2^	19.9 (13.3–34.7)

SD, standard deviation; CI, confidence interval; BMI, body mass index.

**Table 2:** Symptoms reported by patients with abdominal TB (*N* = 29^a^)

Symptoms	*n*	%	95% CI
Weight loss only	3	10%	0–21%
Abdominal pain only	3	10%	0–21%
Weight loss and abdominal pain	5	16.7%	3–31%
Weight loss and diarrhea	3	10%	0–21%
Weight loss and abdominal pain and diarrhea	15	50%	31–69%

CI, confidence interval.

a One patient had only abdominal distension with no further symptoms.

**Table 3:** Laboratory results in patients with abdominal TB

Laboratory test	Value^a^	95% CI
Hb, g/dl	7.4 ± 2.0	
<8 g/dl	65%	45–85%
ALT, U/l	47 ± 33	
>60 U/l	23%	5–41%
GGT, U/l	120 ± 93	
>62 U/l	65%	44–86%
Bilirubin (total), μmol/l	17 ± 20	
>17.0 μmol/l	33%	12–54%
Albumin, g/l	16 ± 3.3	
<32 g/l	100%	95–100%
Total protein, g/l	71 ± 14	
>80 g/l	32%	12–52%

CI, confidence interval; Hb, hemoglobin; ALT, alanine aminotransferase; GGT, gamma-glutamyl transferase.

a Values are mean ± standard deviation.

**Table 4:** Sonographic findings in abdominal TB patients (*N* = 30)

Sonographic findings	*n*	%	95% CI
Lymph nodes only	4	13%	1–25%
Splenic lesions and ascites	1	3%	0–9%
Lymph nodes and ascites	11	37%	19–55%
Lymph nodes and splenic lesions	4	13%	1–25%
Lymph nodes and splenic lesions and ascites	10	33%	16–50%

CI, confidence interval.
